# Acute skin failure knowledge, attitudes and practices amongst intensive care unit nurses in China: A multicentre cross‐sectional survey

**DOI:** 10.1111/iwj.70099

**Published:** 2025-01-12

**Authors:** Xiuru Yang, Fenglin Yan, Sha Xie, Yinju Shang, Mei Wang, Dan Wen, Haiyan He

**Affiliations:** ^1^ Intensive Care Unit, Mianyang Central Hospital, School of Medicine University of Electronic Science and Technology of China Mianyang China

**Keywords:** clinical competence, health knowledge, attitudes, practice, intensive care units, nursing care, skin

## Abstract

**Purpose:**

To investigate the knowledge, attitudes and practices of intensive care unit (ICU) nurses regarding acute skin failure (ASF) and analyse the influencing factors thereof.

**Methods:**

From 22 December 2023 to 24 January 2024, a cross‐sectional study was conducted amongst ICU nurses from 21 hospitals in eight provinces in China. The ASF knowledge, attitude and practice scores were determined using questionnaires, and multiple linear regression was used for further analysis.

**Results:**

Overall, 304 ICU nurses completed the survey. The knowledge, attitude and practice scores were 24.89 ± 10.93, 40.67 ± 5.93 and 43.47 ± 9.19, with scoring rates of 45.25%, 81.34% and 72.45%, respectively. Multiple linear regression analysis showed that being a wound ostomy specialist nurse was positively correlated with the knowledge dimension score (*p* < 0.05). Nurses' professional titles significantly affected attitude scores (*p* < 0.05); the higher the professional title, the more positive the attitude towards ASF.

**Conclusion:**

The attitudes and practices of ICU nurses in China towards ASF were found to be positive; however, their knowledge levels need improvement. Nursing managers should conduct targeted training, especially for entry‐level nurses.

**Abbreviations**
ASF: acute skin failure
ICU: intensive care unit
PI: pressure injury

## 1 INTRODUCTION

The skin, the human body's largest organ, accounts for 10%–15% of body weight, with its blood supply accounting for 25%–33% of the cardiac output.^1^ In 1991, La Puma^2^ proposed that the skin can fail like any other organ. In 2006, Langemo and Brown^3^ defined acute skin failure (ASF) as necrosis of the skin and subcutaneous tissue during acute and critical illness owing to hypoperfusion, haemodynamic instability and decreased tissue tolerance. The clinical manifestations include skin mottling, moist coldness and decreased fingertip blood–oxygen saturation.^4^ Pear, butterfly or horseshoe‐shaped red, purple or yellow‐black ulcers may suddenly appear on the skin over various body parts. Cohen et al. reported that patients with ASF had a mortality rate of 42%.^5^

As the clinical manifestations of ASF are similar to those of pressure injuries (PI), they are often clinically confused. PI is a localized skin/subcutaneous soft tissue injury due to intense and prolonged pressure or pressure combined with shear force.^6^, ^7^ The overall prevalence of PI in critically ill patients is 26.6%.^8^ ASF is a skin tissue injury caused by haemodynamic instability and hypoperfusion in critically ill patients.^4^ The main factor in PI development is extrinsic pressure, whereas the underlying cause of ASF is inadequate blood flow to the skin.^9^

Assessing cutaneous hypoperfusion is critical for early recognition of ASF.^10^ Failing to recognize ASF and its risk factors and not adequately intervening with skin perfusion issues will directly affect the prognosis. Early recognition and correct differentiation between ASF and PI are vital to prevent the development of ASF. Current research on ASF in China has focused on risk factors, assessment and identification. However, nurses' awareness and practices regarding acute skin breakdown still need to be more effective.

We conducted a multicentre, cross‐sectional survey of intensive care unit (ICU) nurses in China, aiming to understand ICU nurses' knowledge, beliefs and behaviours regarding ASF and provide a reference for conducting training.

## 2 METHODS

### 2.1 Participants

We conducted a cross‐sectional survey from 22 December 2023 to 24 January 2024. Convenience sampling was used to select ICU nurses from 21 hospitals in eight provinces in China (Fujian, Gansu, Guangdong, Guangxi, Guizhou, Hunan, Inner Mongolia and Sichuan) as survey respondents. The nurses in this study worked in medical, neonatal, paediatric, cardiac and neurosurgical ICUs.

The inclusion criteria were registered nurses, ICU clinical work time of ≥1 year and voluntary participation. Interns, training nurses and nurses absent during the survey period (e.g., on sick or maternity leave) were excluded. The study protocol was approved by the Ethics Committee of the Mianyang Central Hospital (approval number: S202303119‐01). All the participants provided written informed consent. All the procedures were conducted following the principles of the Declaration of Helsinki.

### 2.2 Questionnaires

#### 2.2.1 Demographic profile questionnaire

The research team designed a demographic questionnaire to assess sex, age, education, marital status, professional title and hospital level, whether they were ICU or wound ostomy specialist nurses, and years of experience in the ICU.

#### 2.2.2 Questionnaire for assessing ICU nurses' ASF knowledge, attitudes and practices

The questionnaire consisting of 33 items was developed by Yang et al.^11^ and included knowledge, attitude and behaviour dimensions; the questionnaire was developed in 2023 based on the Knowledge–Attitude–Practice framework, a well‐established model for understanding and modifying individual behaviours. Yang et al. tested the reliability and validity of the questionnaire, achieving an excellent Cronbach's *α* of 0.941 for internal consistency and a high test–retest correlation of 0.956, demonstrating the substantial homogeneity and stability of the questionnaire. Permission was obtained from the authors to use the questionnaire before conducting the survey. Before conducting the formal investigation, 20 ICU nurses were selected for a preliminary survey. The total content validity of the questionnaire was 0.848, the total Kaiser–Meyer–Olkin value was 0.875 and the total Cronbach's *α* coefficient was 0.941. The Cronbach's *α* coefficients of knowledge, attitude and practices were 0.923, 0.932 and 0.934, respectively.

The knowledge dimension comprised 11 items, including the definition of ASF, its pathogenesis, risk factors, location, clinical manifestations, the relationship between ASF and microperfusion, skin perfusion pressure evaluation methods, tissue perfusion evaluation tools, hypoperfusion improvement measures, ASF prevention measures and ASF nursing measures. A five‐point Likert scale was used for scoring. Scores of 1–5 corresponded to ‘very low knowledge’, ‘low knowledge’, ‘moderate knowledge’, ‘high knowledge’ and ‘very high knowledge’, respectively. The total possible score was 55, with higher scores indicating a better understanding of ASF amongst ICU nurses.

The attitudinal dimension comprised 10 items, including the difference between ASF and PI, the role of ICU nurses in ASF care, the importance of skin perfusion assessment, the multi‐departmental management of ASF and standardized prevention and treatment procedures. A five‐point Likert scale was used for scoring. Scores of 1–5 corresponded to ‘completely disagree’, ‘disagree’, ‘neutral’, ‘agree’ and ‘fully agree’, respectively. The total possible score was 50 points, and the higher the score, the more positive the attitude of the ICU nurses towards ASF.

The behavioural dimension comprised 12 items, including active learning of ASF, assessment of skin perfusion, attention to haemodynamic changes, conscious implementation of ASF nursing and active implementation of ASF prevention and treatment. A five‐point Likert scale was used for scoring. Scores of 1–5 corresponded to ‘never’, ‘occasionally’, ‘sometimes’, ‘often’ and ‘always’, respectively. The total possible score was 60, and the higher the score, the better the behaviour of ICU nurses towards ASF.

The questionnaire's total score combined the knowledge, attitude and practice dimensions scores. The scores ranged from 33 to 165, and the higher the score, the better the cognition level of the ICU nurses regarding ASF. The scoring rate of the questionnaire's total score and the knowledge, attitude and practice dimensions was calculated as the average score/dimension total score × 100%. A scoring rate of >85% was considered high, <60% was considered low and 60%–85% was considered medium.

### 2.3 Data collection and quality control methods

The Wenjuanxing platform was used to distribute and retrieve electronic questionnaires (https://www.wjx.cn/). Before initiating the study, the research team contacted the ICU head nurse at the hospital where the survey was conducted. The head nurse was informed of the purpose and content of the survey; their assistance was sought to disseminate information to potential participants. The link and quick response codes of the questionnaire were released after informed consent was obtained. The questionnaire included an informed consent form and standardized guidelines explaining the study's purpose, content and precautions. When accessing the survey, participants were not required to provide identifying information such as their names, email addresses or other personal details. Questionnaires with answer times of less than 120 s were considered invalid. The same IP address was used only once to prevent duplication.

### 2.4 Statistical analysis

SPSS 26.0 (IBM, Armonk, NY, USA) statistical software was used for data analysis. Categorical variables were described using frequencies and percentages. Continuous variables were expressed as mean ± standard deviation when they conformed to a normal distribution and were analysed using independent sample *t*‐tests or one‐way analysis of variance. Non‐normally distributed continuous variables were expressed as medians and quartiles using the Wilcoxon rank‐sum test. Multiple linear regression was used to analyse the factors affecting ICU nurses' perceptions of ASF. The statistical test level was *α* = 0.05, and a *p*‐value of <0.05 indicated statistical significance.

## 3 RESULTS

### 3.1 Demographic characteristics

A total of 311 questionnaires were distributed, and 304 were returned, with a valid recovery rate of 94.54%. Overall, 304 nurses participated in the survey: 275 (90.5%) from tertiary hospitals and 29 (9.5%) from secondary hospitals. Of the respondents, 259 (85.2%) were women and 45 (14.8%) were men. Of all respondents, 174 (57.3%) were aged 26–35 years and had a bachelor's degree (230, 75.7%); 161 (53.0%) were ICU specialist nurses and 10 (3.3%) were wound ostomy specialist nurses. The demographic characteristics of the participants are presented in Table 1.

**TABLE 1:** The demographic characteristics of the participants (*n* = 304, $\bar{\chi }$ ± s).

Characteristic	Number (%)	Knowledge dimension	Attitude dimension	Practice dimension	Total score of knowledge–attitude–practice
Gender					
Male	45 (14.8)	24.44 ± 12.08	39.93 ± 5.74	41.38 ± 10.43	105.75 ± 22.14
Female	259 (85.2)	24.97 ± 10.74	40.80 ± 5.96	43.83 ± 8.93	109.60 ± 19.16
*t*‐value		−0.299	−0.90	−1.66	−1.214
*p*‐value		0.77	0.37	0.10	0.226
Age					
≤25 years	41 (13.5)	24.95 ± 11.56	39.59 ± 6.35	42.90 ± 9.87	107.43 ± 21.58
26–35 years	174 (57.3)	24.30 ± 10.77	40.35 ± 6.25	42.84 ± 9.10	107.47 ± 19.29
36–45 years	74 (24.3)	25.73 ± 11.01	41.92 ± 4.79	45.00 ± 8.92	112.64 ± 19.28
≥46 years	15 (4.9)	27.53 ± 11.06	41.33 ± 5.21	44.73 ± 9.57	113.60 ± 18.70
*F*‐value		0.61	1.80	1.10	1.569
*p*‐value		0.61	0.15	0.35	0.197
Marital status					
Married	92 (30.3)	22.97 ± 11.32	39.58 ± 6.06	42.89 ± 9.32	105.43 ± 20.38
Unmarried	212 (69.7)	25.73 ± 10.68	41.14 ± 5.82	43.72 ± 9.14	110.59 ± 19.14
*t*‐value		−2.04	−2.13	−0.72	−2.116
*p*‐value		**0.04**	**0.03**	0.47	**0.035**
Education					
Specialty degree	70 (23.0)	25.31 ± 10.62	40.51 ± 5.60	44.73 ± 8.95	110.55 ± 18.76
Bachelor's degree	230 (75.7)	24.82 ± 11.12	40.71 ± 6.04	43.07 ± 9.28	108.57 ± 20.01
Master's degree and above	4 (1.3)	21.75 ± 4.03	42.00 ± 5.60	44.75 ± 7.76	108.50 ± 14.84
*F*‐value		0.22	0.13	0.92	0.273
*p*‐value		0.80	0.88	0.40	0.762
Professional title					
Nurse	58 (19.1)	26.38 ± 11.08	38.50 ± 6.52	41.50 ± 10.09	106.37 ± 20.52
Nurse practitioner	109 (35.8)	24.23 ± 10.77	40.12 ± 5.93	42.67 ± 8.38	107.01 ± 19.42
Supervisor nurse	117 (38.5)	24.85 ± 11.19	42.01 ± 5.38	44.87 ± 9.19	111.71 ± 19.05
Associate chief nurse and above	20 (6.6)	24.45 ± 10.19	42.20 ± 5.03	45.35 ± 9.72	112.00 ± 20.62
*F*‐value		0.50	5.61	2.38	1.626
*p*‐value		0.68	**<0.01**	0.07	0.183
Years of experience in ICU					
1–5 years	141 (46.4)	24.63 ± 11.06	40.38 ± 5.91	43.09 ± 9.24	108.07 ± 19.56
6–10 years	81 (26.6)	23.26 ± 10.46	39.44 ± 6.76	42.83 ± 9.69	105.53 ± 20.16
11–15 years	57 (18.8)	26.14 ± 11.34	42.68 ± 4.26	44.58 ± 7.99	113.40 ± 18.57
16–20 years	15 (4.9)	26.53 ± 8.64	41.20 ± 5.53	46.07 ± 9.07	113.80 ± 14.82
≥21 years	10 (3.3)	32.30 ± 11.47	42.70 ± 4.92	43.90 ± 11.37	118.90 ± 23.13
*F*‐value		1.91	3.00	0.67	2.326
*p*‐value		0.11	**0.02**	0.61	0.056
Hospital level					
Tertiary hospital	275 (90.5)	24.88 ± 11.06	40.69 ± 6.06	43.47 ± 9.29	109.03 ± 20.15
Secondary hospital	29 (9.5)	25.17 ± 10.01	40.52 ± 4.76	43.79 ± 8.21	109.48 ± 14.35
*F*‐value		0.02	0.02	0.03	0.013
*p*‐value		0.89	0.88	0.86	0.908
ICU specialist nurses					
Yes	161 (53.0)	24.89 ± 10.52	40.50 ± 5.75	43.10 ± 8.65	108.49 ± 18.89
No	143 (47.0)	24.90 ± 11.41	40.86 ± 6.14	43.89 ± 9.77	109.64 ± 20.49
*t*‐value		−0.001	−0.53	−0.75	−0.510
*p*‐value		1.00	0.60	0.46	0.610
Wound ostomy specialist nurses					
Yes	10 (3.3)	33.30 ± 11.63	41.50 ± 5.85	46.60 ± 4.60	121.40 ± 17.91
No	294 (96.7)	24.61 ± 10.81	40.64 ± 5.94	43.36 ± 9.29	108.61 ± 19.58
*t*‐value		2.494	0.45	2.09	2.035
*p*‐value		**0.013**	0.65	0.06	**0.043**

*Note*: The bold values indicate statistical significance at *P* < 0.05.

Abbreviation: ICU, intensive care unit.

### 3.2 ICU nurse ASF knowledge, attitude and practice scores

The total score of ICU nurses' knowledge, attitudes and practices towards ASF was 109.03 ± 19.64, and the scoring rate was 66.07%. The average knowledge, attitude and practice dimension scores were 24.89 ± 10.93, 40.67 ± 5.93 and 43.47 ± 9.19, with 45.25%, 81.34% and 72.45% scoring rates, respectively. The items with the lowest scores are listed in Table 2.

**TABLE 2:** The three items with the lowest scores on knowledge, attitude and practice of acute skin failure of intensive care unit (ICU) nurses.

Dimension	Item	Score($\bar{\chi }$ ± s)
Knowledge dimension	1. Do you know the evaluation method of skin perfusion pressure?	2.00 ± 1.12
	2. Do you know the evaluation tools for tissue perfusion?	2.10 ± 1.16
	3. Do you know the pathogenesis of acute skin failure?	2.21 ± 1.07
Attitude dimension	1. Do you think acute skin failure is an inevitable condition for critically ill patients?	3.85 ± 0.82
	2. Do you think acute skin failure is different from pressure injury?	3.88 ± 0.77
	3. Do you think it is important to routinely evaluate skin perfusion in ICU patients?	4.05 ± 0.72
Practice dimension	1. Do you assess patients for skin perfusion?	3.02 ± 1.19
	2. Will you implement warming measures for patients with acute skin failure?	3.29 ± 0.98
	3. Would you be willing to initiate training related to acute skin failure?	3.29 ± 1.07

### 3.3 Comparing ICU nurses' ASF knowledge, attitude and practice scores with different characteristics

General demographic information was used as the independent variable to analyse the differences in knowledge, attitudes and practices regarding ASF amongst nurses with different characteristics. The univariate analysis showed significant differences in the total and knowledge questionnaire scores of ICU nurses with varying levels of hospital, marital status and wound ostomy specialist nurses (*p* < 0.05). Significant differences were also observed in attitude scores amongst the hospital levels, marital status, professional titles and work experience in the ICU (*p* < 0.05). The practice scores also showed significant differences at different hospital levels (*p* < 0.05) (Table 1).

### 3.4 Multivariate linear regression analysis of ICU nurse ASF knowledge, attitudes and practices

Statistically significant variables in the univariate analysis were used as independent variables. The regression analysis used knowledge, attitude and practice scores as dependent variables. Table 3 presents the values assigned to the independent variables. Multiple linear regression analysis showed that being a wound ostomy specialist nurse was significant regarding the knowledge dimension score (*p* < 0.05). Professional titles significantly affected the attitude dimension score (*p* < 0.05). ASF knowledge positively correlated with being a specialist nurse in wound ostomies. The higher the professional title, the more positive the attitude towards ASF. The details of the multiple linear regressions are presented in Table 4.

**TABLE 3:** Independent variable assignment table.

Independent variable	Assignment method
Hospital level	Tertiary hospital = 0, secondary hospital = 1
Marital status	Unmarried = 0, married = 1
Professional title	Nurse = 0, nurse practitioner = 1, supervisor nurse = 2, associate chief nurse and above = 3
Wound ostomy specialist nurses	Yes = 0, no = 1
Years of experience in intensive care unit	1–5 years = 0, 6–10 years = 1, 11–15 years = 2, 16–20 years = 3, ≥21 years = 4

**TABLE 4:** Multivariate linear regression analysis of knowledge, attitude and practice of intensive care unit (ICU) nurses on acute skin failure.

Dependent variable	Independent variable	Regression weight	Standard error	Standardized regression weights	*t*‐value	*p*‐value
Knowledge dimension^a^	Constant	30.850	4.279		7.209	<0.001
	Hospital level	0.302	2.113	0.008	0.143	0.886
	Marital status	2.647	1.353	0.111	1.956	0.051
	Wound ostomy specialist nurses	−8.397	3.483	−0.137	−2.411	**0.017**
Attitude dimension^b^	Constant	36.716	1.674		21.930	<0.001
	Hospital level	0.256	1.150	0.013	0.222	0.824
	Marital status	0.449	0.823	0.035	0.546	0.586
	Professional title	1.632	0.533	0.235	3.065	**0.002**
	Years of experience in ICU	−0.230	0.418	−0.041	−0.550	0.583
Practice dimension^c^	Constant	43.156	2.038		21.177	<0.001
	Professional title	0.319	1.796	0.010	0.177	0.859

^a^ *R* ^2^ = 0.033, Adjusted *R* ^2^ = 0.023, *F* = 3.356, *p* = 0.019.

^b^ *R* ^2^ = 0.051, Adjusted *R* ^2^ = 0.039, *F* = 4.031, *p* = 0.003.

^c^ *R* ^2^ = 0.000, Adjusted *R* ^2^ = ‐0.003, *F* = 0.031, *p* = 0.859.

## 4 DISCUSSION

In this study, ICU nurses scored 24.89 ± 10.93 on the ASF knowledge dimension, with a scoring rate of 45.25%, classified as a low level. This observation aligns with the results reported by Yang et al., further reinforcing the notion that there is a notable deficiency in understanding ASF amongst ICU nurses.^11^ The consistency of our findings demonstrates the need for targeted interventions and educational programmes to enhance ICU nurses' knowledge bases in this critical area. The three items with the lowest scores in the knowledge dimension included the pathogenesis of ASF, the evaluation method of skin perfusion pressure and the tool for tissue perfusion. This indicates that ICU nurses do not know enough about ASF to correctly assess it in clinical practice, which poses safety risks. No uniform criteria exist for defining and diagnosing ASF.^12^ As a special type of skin injury, nurses often confuse it with PI, leading to a delayed response, as patients cannot receive timely and proper treatment. PI is defined as poor local circulation under the combined effects of prolonged high pressure and shear forces, resulting in ischaemia and hypoxia.^13^ Recent studies suggested that ASF is secondary to acute disease‐related malperfusion.^14^ The two differ in etiological mechanisms, location, evaluation methods and susceptible populations.^15^ Evaluation of skin hypoperfusion status is essential for the early detection of ASF. The skin speckle score and skin perfusion pressure monitoring are effective evaluation methods.^10^ When shock occurs, peripheral skin capillaries constrict, affecting microcirculation and causing symptoms such as cold, clammy and florid skin.^16^ Skin perfusion pressure effectively evaluates changes in skin and subcutaneous tissue microcirculation.^17^ Laser Doppler is essential for determining the degree of ischaemia and the prognosis of limb ischaemic ulcers and wounds.^18^

The total score of ICU nurses' attitude towards ASF was 40.67 ± 5.93, with a scoring rate of 81.34%, classified as medium level. The results showed that the ICU nurses had a positive attitude towards ASF. Notably, our results align with the previous study conducted by Fu et al.^19^ They found that nurses hold a favourable perspective towards ASF. However, their level of knowledge and practical application in this domain necessitated substantial enhancement. The items with the lowest scores in the attitude dimension included skin failure in critically ill patients, differences between ASF and PI and routine assessment of skin perfusion. Studies have shown that mechanical ventilation, respiratory failure and sepsis are risk factors for ASF.^20^ Critically ill patients are at high risk of developing ASF. Although ICU nurses were aware of the importance of prevention, they lacked knowledge about ASF. It is challenging to distinguish ASF from stress injury and identify the risk factors promptly.^21^ Differences from stress injuries must be evaluated in terms of perception, mobility, nutritional status, skin moisture, friction and shear force.^22^ ASF is associated with skin perfusion. Skin perfusion pressure is an essential index for measuring the microcirculation in human tissues. Values lower than 25–30 mmHg led to local skin and subcutaneous tissue ischaemia, necrosis and skin failure.^23^

The ASF practice dimension score was 43.47 ± 9.19, with a scoring rate of 72.45%, classified as medium level, indicating that ICU nurses should focus on critically ill patients and implement targeted intervention measures. The three items with the lowest scores in the practice dimension were skin perfusion assessment, implementation of warmth measures and active knowledge learning. Haemodynamic instability is a significant cause of skin failure.^4^ ICU nurses can adjust vasoactive drugs based on haemodynamics after communicating with physicians. However, they did not understand the mechanism of skin failure or the severe impact of abnormal haemodynamics on patients. The peripheral perfusion index is a noninvasive index for assessing circulation. When the index is less than 1.4, the body tissue is hypoperfused, which can quickly induce ASF.^4^ Nurses mainly focused on indicators such as heart rate, blood pressure and urine volume without considering the skin as an organ for evaluating perfusion.^24^

Our results showed that the knowledge dimension was affected by whether the nurses were wound ostomy specialists (*p* < 0.05), and the attitude dimension was affected by professional titles (*p* < 0.05). Wound ostomy specialist nurses are distinguished from ICU specialists by their greater specialization and proficiency in managing skin problems. Entry‐level nurses require additional knowledge and clinical experience to address complex issues. Nursing managers should conduct stratified training, and wound stomatology nurses should focus more on ASF. Guidance should be provided to clinical nurses to improve their ability to recognize and treat ASF.

### 4.1 Limitations

This study had some limitations. First, it adopted a convenience sampling method, and the survey participants were limited to secondary‐ and tertiary‐level hospitals in eight provinces in China. Therefore, the sample may have been underrepresented. Second, the data were collected using self‐report questionnaires, which could present a risk of reporting bias. Future research should adopt a more objective evaluation method and conduct a large‐scale nationwide survey to better understand ICU nurses' knowledge levels regarding ASF.

## 5 CONCLUSIONS

The attitudes and practices of ICU nurses in China towards ASF were positive; however, their knowledge level needs improvement. ICU nurses' knowledge, attitudes and practices regarding ASF were influenced by their professional titles and whether they were wound ostomy nurses. Nursing managers should conduct targeted training, especially for entry‐level nurses. Various training methods should be used to enhance nurses' awareness of ASF. By improving the level of ASF knowledge amongst nurses, relevant practices can be implemented to promote patient safety and quality of care in the ICU.

## CONFLICT OF INTEREST STATEMENT

The authors declare no conflict of interest.

## ETHICS STATEMENT

This study was conducted in accordance with the declaration of Helsinki. The study protocol was approved by the Ethics Committee of Mianyang Central Hospital (approval number: S202303119‐01).

## Data Availability

Data are available upon request from the authors.
